# Cervical human papillomavirus infection in women with preterm prelabor rupture of membranes

**DOI:** 10.1371/journal.pone.0207896

**Published:** 2018-11-21

**Authors:** Helena Hornychova, Marian Kacerovsky, Ivana Musilova, Lenka Pliskova, Helena Zemlickova, Adela Matejkova, Hana Vosmikova, Katerina Rozkosova, Petra Cermakova, Radka Bolehovska, Petr Halada, Bo Jacobsson, Jan Laco

**Affiliations:** 1 The Fingerland Department of Pathology, University Hospital Hradec Kralove, Charles University, Faculty of Medicine in Hradec Kralove, Hradec Kralove, Czech Republic; 2 Department of Obstetrics and Gynecology, University Hospital Hradec Kralove, Charles University, Faculty of Medicine in Hradec Kralove, Hradec Kralove, Czech Republic; 3 Biomedical Research Center, University Hospital Hradec Kralove, Hradec Kralove, Czech Republic; 4 Institute of Clinical Biochemistry and Diagnostics, University Hospital Hradec Kralove, Charles University, Faculty of Medicine in Hradec Kralove, Hradec Kralove, Czech Republic; 5 Department of Microbiology, University Hospital Hradec Kralove, Charles University, Faculty of Medicine in Hradec Kralove, Hradec Kralove, Czech Republic; 6 Department of Obstetrics and Gynecology, Sahlgrenska Academy, Gothenburg University, Gothenburg, Sweden; 7 Department of Genetics and Bioinformatics, Domain of Health Data and Digitalisation, Institute of Public Health, Oslo, Norway; Universidade Estadual de Maringa, BRAZIL

## Abstract

**Objective:**

To evaluate the association between cervical human papillomavirus (HPV) infection at the time of admission and the presence of microbial invasion of the amniotic cavity (MIAC) and intra-amniotic inflammation (IAI) in women with preterm prelabor rupture of membranes (PPROM) and to determine the association between cervical HPV infection and short-term neonatal morbidity.

**Methods:**

One hundred women with singleton pregnancies complicated by PPROM between the gestational ages of 24+0 and 36+6 weeks were included in the study. The presence of HPV DNA was evaluated in scraped cervical cells using polymerase chain reaction (PCR). Amniotic fluid samples were obtained by transabdominal amniocentesis.

**Results:**

The rate of cervical HPV infection in women with PPROM was 24%. The rates of MIAC and IAI were not different between women with cervical HPV infection and those without cervical HPV infection [MIAC: with HPV: 21% (5/24) vs. without HPV: 22% (17/76), *p* = 1.00; IAI: with HPV: 21% (5/24) vs. without HPV: 18% (14/76), *p* = 0.77]. There were no differences in the selected aspects of short-term neonatal morbidity between women with and without cervical HPV infection.

**Conclusions:**

In women with PPROM, the presence of cervical HPV infection at the time of admission is not related to a higher risk of intra-amniotic infection-related and inflammatory complications or worse short-term neonatal outcomes.

## Introduction

Preterm prelabor rupture of membranes (PPROM), defined as the rupture of fetal membranes and leakage of amniotic fluid before the onset of regular uterine activity prior to a gestational age of 37 weeks, is responsible for about one-third of preterm deliveries and complicates approximately 2–8% of all pregnancies [1–3].

Although PPROM has been considered a non-infectious disease, many PPROM pregnancies are still complicated by infection-related and inflammatory conditions such as microbial invasion of the amniotic cavity (MIAC) and/or intra-amniotic inflammation (IAI) [4–7]. These complications can be considered as gestational age-dependent, since their presence decreases with advancing gestational age of development of PPROM [6, 7]. However, despite extensive research on intra-amniotic complications in PPROM, it is still not known whether these are causes or consequences of PPROM. In addition, there is a lacuna in the knowledge regarding which underlying conditions are responsible for the presence or absence of these complications in PPROM pregnancies. It is likely that the microbial composition of the choriodecidual, cervical, and vaginal compartments plays an important role in the development of intra-amniotic complications [8–11]. For example, the presence of *Lactobacillus crispatus* as a dominant bacterium in the cervical compartment has a protective effect against the development of intra-amniotic complications [9].

Aside from this theory, the presence of viral infection in the pregnant cervix in a murine model has been recently shown to be a critical factor that may dramatically reduce the capacity of the cervix to prevent ascent of microorganisms from the vagina to the amniotic cavity [12]. Cervical viral infection affects the expression and function of toll-like receptors (receptors recognizing bacteria and non-self molecules) and antimicrobial peptides [12]. In addition, a preexisting viral infection of the placenta might amplify the inflammatory response of the placenta to local microorganisms [12–14].

In pregnant women, the most common viral cervical infection is caused by human papillomavirus (HPV) with prevalence varying between 6–67% [15–18]. It is indisputable that HPV as an oncovirus has the potential to cause cervical carcinoma with faster progression from cervical high-grade squamous intraepithelial lesion into cancer during the pregnancy [19–22]. On the other hand, the association between HPV cervical infection and spontaneous preterm birth has not been fully elucidated due to conflicting results from available studies [16, 23–25]. It has not been established whether cervical HPV infection may predispose to ascent of bacteria from the lower genital tract to the amniotic cavity and lead to the development of infection-related and inflammatory intra-amniotic complications.

Therefore, the main aim of the study was to evaluate the association between cervical HPV infection at the time of admission and the presence of intra-amniotic infection-related and inflammatory complications in women with PPROM between gestational ages 24+0 and 36+6 weeks. The secondary aim of the study was to determine the association between cervical HPV infection at the time of admission and short-term neonatal morbidity.

## Material and methods

A prospective cohort study of women with singleton pregnancies complicated by PPROM between gestational ages 24+0 and 36+6 weeks who were admitted to the Department of Obstetrics and Gynecology, University Hospital Hradec Kralove between January 2016 and January 2017, was conducted. Women who were at least 18 years old were included in the study. Women with pregnancies complicated by the presence of diabetes mellitus, gestational diabetes mellitus, preeclampsia, pregnancy-induced hypertension, chronic hypertension, the presence of chromosomal or structural fetal abnormalities, fetal growth restriction, signs of fetal hypoxia or vaginal bleeding were excluded from the study.

**Fig 1:**
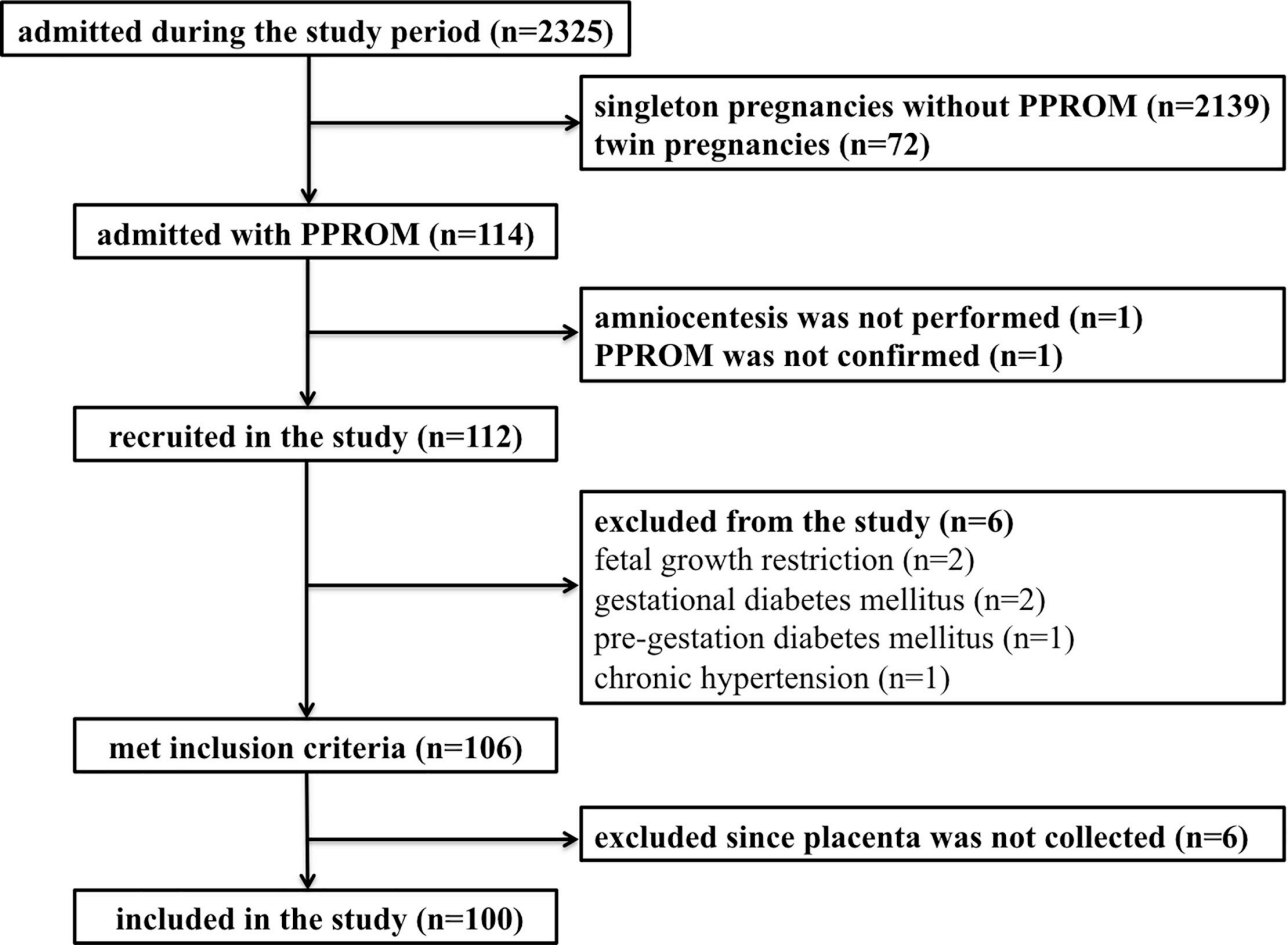
Flow diagram describing selection of women participants.

In total, 2325 pregnant women were admitted at the delivery unit during the study period, 114 out of them were women with singleton pregnancies complicated with PPROM at gestational ages between 24+0 and 36+6 weeks. Two women were not eligible owing to amniocentesis failure and delivery before the amniocentesis (n = 1 each). Therefore, 112 women with PPROM were enrolled in the study. Six women were excluded because of medical reasons: fetal growth restriction (n = 2), gestational diabetes mellitus (n = 2), and pre-gestation diabetes mellitus and chronic hypertension (n = 1 each). Subsequently, 6 women were excluded due to missing results because the placentas had not been collected after delivery. Finally, 100 women were included in the study (Fig 1). None of the included women was vaccinated against HPV, had a pre-invasive cervical lesions prior to the current pregnancy, underwent cervical conisation or amputation.

Gestational ages were established based on the first-trimester fetal biometry. Women with PPROM between gestational ages 24+0 and 34+6 weeks were given antibiotics and corticosteroids to accelerate lung maturation, whereas antibiotic treatment alone was provided to women with PPROM beyond gestational age 34+6 weeks. Women with the presence of both amniotic fluid IL-6 ≥745 pg/mL and the presence of MIAC beyond 28 gestational weeks were actively managed. In actively managed women, labor was induced or an elective cesarean section was performed within 72 h of admission to the delivery unit. The remaining women with PPROM were managed expectantly.

PPROM was confirmed by examining the women using a sterile speculum to verify the pooling of amniotic fluid in the vagina. If clinical PPROM was unconfirmed, the leakage of amniotic fluid was diagnosed by the presence of insulin-like growth factor binding proteins (Actim PROM test; Medix Biochemica, Kauniainen, Finland) in the vaginal fluid.

This study’s protocol was approved by the Ethics Committee of the University Hospital of Hradec Kralove, Czech Republic (July 2014; No 201407 S14P). All women provided written informed consent and were self-reported as Caucasians. Amniotic, cervical, vaginal, and crevicular samples as well as umbilical cord blood samples from some women in this cohort were used in our previously published studies [8, 26–31].

### Sample collection

Cervical specimens were taken with the use of a cervical brush (Rovers Medical Devices, KV Oss, Netherlands) from the endocervical canal immediately before amniocentesis. The cervical brush was detached and suspended in a liquid-based cytology vial (DiaPrep Fixative Gyn, Diapath S.P.A, Martinengo, Italy) containing preservative fluid and taken immediately to the cytopathology laboratory for further processing.

Ultrasound-guided transabdominal amniocentesis was performed upon admission before the administration of corticosteroids and antibiotics; approximately 1–2 mL of amniotic fluid was obtained. Upon collection, amniotic fluid samples were processed as described earlier [29, 30, 32]. A total of 100 μL of non-centrifuged amniotic fluid was used for the bedside assessment of interleukin (IL)-6 concentration. Two aliquots of non-centrifuged amniotic fluid were immediately transported to the microbiology laboratory for polymerase chain reaction (PCR) testing for *Ureaplasma* species, *Mycoplasma hominis*, and *Chlamydia trachomatis* and the 16S rRNA gene, as well as for aerobic/anaerobic cultivation of amniotic fluid.

After delivery, the placenta, fetal membranes, and the umbilical cord were fixed in 10% neutral buffered formalin. Tissue samples were obtained from the placenta (at least 2 samples and a special block with 3 narrow samples from basal decidua), fetal membranes (1 sample from free margin of membranes, 1 from the central part of membranes, and 1 from membranes with a marginal part of the placenta), and umbilical cord (usually 1 sample). Tissue samples were routinely processed and embedded in paraffin. The sections of tissue blocks were stained with hematoxylin and eosin. Four blocks (2 with the placenta, 1 with free fetal membranes, and 1 with basal decidua and chorionic villi) from each placenta were used for further analysis of HPV status.

### Liquid-based cervical cytology

A cytology slide was produced from each liquid-based cytology sample using a Rotofix 32 A centrifuge (Hettich, Tuttlingen, Germany). The slides were analyzed with the Bethesda Classification System by an experienced cytopathologist (P.C.), who was blinded to the study data.

### HPV detection and genotyping

DNA was extracted from cell suspension using the MagCore Viral Nucleic Acid Extraction Kit (RBC Bioscience, Taiwan), according to the manufacturer´s protocol.

DNA was extracted from the paraffin-embedded placental and fetal membrane tissue with the MagCore Genomic DNA FFPE One-Step Kit (RBC Bioscience, Taiwan), according to the manufacturer´s protocol.

HPV DNA detection and genotyping was performed using qualitative real-time PCR with the AmoyDx Human Papillomavirus Genotyping Detection Kit (Amoy Diagnostics, China). The test has been designed for specific amplification of L1 gene in HPV DNA to detect and genotype 19 high-risk HPVs (HPV16, 18, 26, 31, 33, 35, 39, 45, 51, 52, 53, 56, 58, 59, 66, 68, 70, 73, and 82) and 2 low-risk HPVs (HPV 6 and 11). The test was sensitive to a level of 100 copies of HPV DNA per reaction. An internal control in the assay was provided to test for sample quality and the presence of inhibiting factors.

### p16/Ki-67 dual-stained cytology

For p16/Ki-67 dual staining, an additional cytology slide was produced from each liquid-based cytology sample using a Rotofix 32 A centrifuge. The staining was performed manually using a commercial kit specifically designed for simultaneous detection of p16 and Ki-67 in cervical cytology samples (CINtec PLUS Cytology Kit, Roche mtm laboratories AG, Heidelberg, Germany), according to the instructions of the manufacturer, as described previously [33], followed by hematoxylin counterstaining. Each staining run included control specimens. Slides were analyzed and scored by an experienced cytopathologist (J.L.), who was blinded to all other study data. Samples were considered p16/Ki-67 dual-stain-positive when immunoreactivity for both p16 and Ki-67 was detected within the same cell (i.e., cytoplasmic brown staining for p16, together with nuclear red staining for Ki-67), in at least 1 cell per slide, irrespective of morphology [33].

### Definition of IAI

IAI in PPROM pregnancies was defined as amniotic fluid bedside IL-6 concentrations of 745 pg/mL and higher [34, 35].

### Diagnosis of MIAC

MIAC was confirmed based on a positive PCR analysis result for *Ureaplasma* species, *M*. *hominis* or *C*. *trachomatis;* and/or positivity for the 16S rRNA gene and/or positive aerobic and anaerobic cultivation results from the amniotic fluid.

### Diagnosis of histologic chorioamnionitis

The degree of polymorphonuclear leukocyte infiltration was assessed separately in the free membranes (amnion and chorion-decidua), in the chorionic plate, and in the umbilical cord according to the criteria given by Salafia et al. [36]. Diagnosis of histologic chorioamnionitis (HCA) was based on the presence of inflammatory changes in the chorion-decidua (grades 3–4), chorionic plate (grades 3–4), umbilical cord (grades 1–4), and/or amnion (grades 1–4). Diagnosis of funisitis was based on the presence of inflammatory changes in the umbilical cord (grades 1–4) [36].

### Diagnosis of severe neonatal morbidity

Maternal and perinatal medical records were reviewed by 3 investigators (MK, IM, PH). Data regarding short-term neonatal morbidity were reviewed for all newborns. “Compound neonatal morbidity” was defined in this study as follows: the need for intubation, and/or the need for nasal continuous positive airway pressure, and/or respiratory distress syndrome (defined by the presence of 2 or more of the following criteria: evidence of respiratory compromise, a persistent oxygen requirement for more than 24 hours, administration of exogenous surfactant, radiographic evidence of hyaline membrane disease); and/or transient tachypnea of the newborn (defined as any oxygen supplemental requirement during the first 6 hours that does not increase during the subsequent 18 hours, improvement in clinical conditions within 3–6 hours, and chest radiographs that are either normal or show reduced translucency, infiltrates, and hyperinflation of the lungs); and/or bronchopulmonary dysplasia (defined as infant oxygen requirement at 28 days of age); and/or pneumonia (diagnosed by abnormal findings on chest X-rays); and/or retinopathy of prematurity (identified using retinoscopy); and/or intraventricular hemorrhage (with diagnosis made using cranial ultrasound examinations according to the procedure of Papile et al. [37]); and/or necrotizing enterocolitis (defined as radiologic findings of either intramural gas or free intra-abdominal gas); and/or early- (during the first 72 hours of life) or late-onset (between the ages of 4 and 120 days) sepsis (either proven by bacterial culture or clinically strongly-suspected sepsis); and/or neonatal death before hospital discharge.

### Statistical analysis

The demographic and clinical characteristics were compared using the nonparametric Mann-Whitney *U* test and presented as medians [interquartile range (IQR)] for continuous variables. Categorical variables were compared using Fisher’s exact test or chi-square test, as appropriate, and were presented as numbers (%). The normality of the data was tested using the D’Agostino and Pearson omnibus normality test. Amniotic fluid IL-6 concentrations were compared using the Mann-Whitney *U* test or Kruskal-Wallis test, as appropriate. The rates of short-term neonatal morbidity were compared using Fisher’s exact test. Differences were considered statistically significant at *p*<0.05. All *p*-values were from 2-tailed tests, and all statistical analyses were performed using SPSS 19.0 for Mac OS X (SPSS Inc., Chicago, IL, USA) and with GraphPad Prism 6.0h for Mac OS X (GraphPad Software, La Jolla, CA, USA).

## Results

### Demographic and clinical characteristics of the study population

The presence of MIAC and IAI was identified in 22% (22/100) and 19% (19/100) of the women, respectively. The microbial isolates from amniotic fluid were as follows: *Ureaplasma* spp.: 12, *C*. *trachomatis*: 1, *Streptococcus agalactiae*: 1, *Parvimonas micra*: 1, *Sneathia sanguinegens*: 1, *Haemophilus influenzae*: 1, *Fusobacterium nucleatum*: 1, *Ureaplasma* spp. + *M*. *hominis*: 1, *Ureaplasma* species + *C*. *trachomatis*: 1, *Ureaplasma* spp. + *Enterococcus faecium*: 1, and *Lactobacillus gasseri* and *Bifidobacterium breve*: 1.

In total, 24% (24/100) of women with PPROM had HPV cervical infection at the time of admission. The maternal and clinical characteristics of the study group according to the presence or absence of cervical HPV infection are shown in Table 1. Women with cervical HPV infection were younger and had a higher rate of smokers than women without cervical HPV infection (*p* = 0.03 and *p* = 0.04, respectively).

**Table 1:** Maternal and clinical characteristics of pregnancies complicated by preterm prelabor rupture of membranes with respect to the presence of cervical HPV infection at the time of admission.

Characteristic	with HPV cervical infection (n = 24)	without HPV cervical infection (n = 76)	*p-*value
Maternal age [years, median (IQR)]	28 (26–30)	30 (27–35)	**0.03**
Primiparous [number (%)]	15 (63%)	49 (65%)	1.00
Pre-pregnancy body mass index [kg/m^2^, median (IQR)]	22.7 (19.3–26.7)	22.4 (20.3–28.1)	0.96
Smoking [number (%)]	7 (29%)	9 (11%)	**0.04**
Interval between PPROM and amniocentesis [hours, median (IQR)]	4 (4–7)	5 (3–9)	0.84
Gestational age at admission [weeks, median (IQR)]	34+2 (33+0–35+2)	35+1 (32+5–36+1)	0.18
Gestational age at delivery [weeks, median (IQR)]	35+0 (33+5–35+4)	35+2 (33+3–36+3)	0.37
Latency between PPROM and delivery [hours, median (IQR)]	23 (12–100)	35 (14–81)	0.68
CRP levels at admission [mg/L, median (IQR)]	4.4 (3.0–10.2)	4.1 (2.2–7.6)	0.46
WBC count at admission [x10^9^ L, median (IQR)]	12.7 (11.9–14.8)	12.0 (10.3–14.5)	0.19
Administration of antibiotics [number (%)]	24 (100%)	75 (99%)	1.00
Administration of corticosteroids [number (%)]	14 (58%)	37 (49%)	0.49
Spontaneous vaginal delivery [number (%)]	19 (79%)	54 (71%)	0.60
Forceps delivery [number (%)]	2 (8%)	1 (1%)	0.14
Cesarean delivery [number (%)]	3 (13%)	21 (28%)	0.17
Birth weight [grams, median (IQR)]	2315 (1965–2640)	2305 (1953–2625)	0.81
Apgar score <7; 5 minutes [number (%)]	0 (0%)	3 (4%)	1.00
Apgar score <7; 10 minutes [number (%)]	0 (0%)	1 (1%)	1.00
Histological chorioamnionitis [number (%)]	63% (15/24)	55% (42/76)	0.64
Funisitis [number (%)]	33% (8/24)	32% (24/76)	0.11

Abbreviations:

HPV: human papillomavirus

CRP: C-reactive protein

WBC: white blood cells

Continuous variables were compared using a nonparametric Mann-Whitney *U* test. Categorical variables were compared using the Fisher’s exact test. Statistically significant results are marked in bold. Continuous variables are presented as median (interquartile range [IQR]) and categorical as number (%).

HPV types 16 and 33 were the most common (n = 6 each). The presence of a single HPV type was revealed in 18% of women [(18/100); 11, 31, 39, 51, 52, 56, 66, 82 (n = 1 each), 33 (n = 4), and 16 (n = 5)]. Two or 3 HPV types were identified in 4% [4/100; 16+33 (n = 1), 31+66 (n = 1), 33+35 (n = 1), and 52+55 (n = 1)] and in 2% [2/100; 18+39+52 and 31+39+68 (n = 1 each)] of women, respectively. Clinical characteristics of the women with cervical HPV infection are shown in Table 2. One women with cervical HPV infection (HPV type 33) had a low-grade squamous intraepithelial lesion in cervical cytology at the time of admission and were positive for p16/Ki67. Remaining women with cervical HPV infection had cervical cytology at the time of admission negative for intraepithelial lesion or malignancy and for p16/Ki67. None of the women with HPV cervical infection had HPV DNA in the placental tissue.

**Table 2:** Clinical characteristics of women with PPROM and cervical HPV infection at the time of admission.

Women	HPV type(s)	AF IL-6 (pg/mL)	MIAC	Placental HPV infection	HCA	Funisitis
1.	11	4752	No	No	Yes	No
2.	16	82	*Ureaplasma* spp.	No	Yes	Yes
3.	16	298	No	No	No	No
4.	16	406	No	No	Yes	No
5.	16	118	No	No	No	No
6.	16	148	No	No	Yes	No
7.	31	114	No	No	Yes	Yes
8.	33	10000	*Sneathia sanguinegens*	No	Yes	Yes
9.	33	107	No	No	Yes	No
10.	33	250	No	No	Yes	Yes
11.	33	268	No	No	No	No
12	39	207	No	No	No	No
13.	51	50	No	No	Yes	Yes
14.	52	50	No	No	No	No
15.	56	336	No	No	No	No
16.	66	278	No	No	No	No
17.	66	351	No	No	No	No
18.	82	244	No	No	No	No
19.	16, 33	10000	*Haemophilus influenzae*	No	Yes	Yes
20.	31, 66	371	No	No	Yes	Yes
21.	33,35	1203	*Ureaplasma* spp., *Enterococcus faecium*	No	Yes	Yes
22.	52,59	168	No	No	Yes	No
23.	18,39,52	3848	*Parvimonas micra*	No	Yes	No
24.	31,39,68	355	No	No	Yes	No

Abbreviations:

HPV: human papilloma virus

AF IL-6: amniotic fluid interleukin-6

MIAC: microbial invasion of the amniotic cavity

HCA: acute histological chorioamnionitis

### HPV cervical infection at the time of admission and intra-amniotic infection-related and inflammatory complications

**Fig 2:**
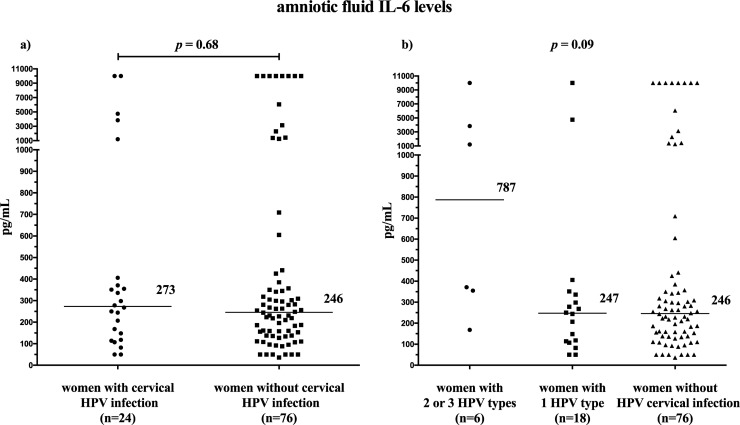
Amniotic fluid IL-6 levels based on the presence or absence of cervical HPV infection (a). Amniotic fluid IL-6 levels based on the presence of 2 or 3 HPV types or 1 HPV type and the absence of cervical HPV infection (b). Abbreviations: IL: interleukin, HPV: human papillomavirus.

Women with HPV cervical infection did not have different rates of MIAC and IAI than women without this complication [MIAC: with HPV: 21% (5/24) vs. without HPV: 22% (17/76), *p* = 1.00; IAI: with HPV: 21% (5/24) vs. without HPV: 18% (14/76), *p* = 0.79]. No difference in amniotic fluid IL-6 levels was found between women with and without cervical HPV infection (with HPV: median 273 pg/mL, IQR 126–397 vs. without HPV: median 246 pg/mL, IQR 138–378; *p* = 0.68; Fig 2).

When women with HPV cervical infection were divided into 2 subgroups based on the number of HPV types present (1 HPV type or 2 or 3 HPV types), no differences in the rates of MIAC and IAI were found among the subgroups [MIAC: with 2 or 3 HPV types: 50% (3/6) vs. with 1 HPV type: 11% (2/18) vs. without HPV: 22% (17/76); *p =* 0.14; IAI: with 2 or 3 HPV types: 50% (3/6) vs. with 1 HPV type: 11% (2/18) vs. without HPV: 18% (14/76); *p =* 0.11). No difference in amniotic fluid IL-6 levels was found among the subgroups (with 2 or 3 HPV types: median 787 pg/mL, IQR 308–5386 vs. with 1 HPV type: median 247 pg/mL, IQR 112–340 vs. without HPV: median 246 pg/mL, IQR 138–378; *p* = 0.09; Fig 2B).

### Cervical HPV infection and selected aspects of short-term neonatal morbidity

No differences in the evaluated aspects of short-term neonatal morbidity were revealed between the newborns from PPROM pregnancies with and without cervical HPV infection at the time of admission (Table 3).

**Table 3:** Short-term neonatal morbidity in newborns from PPROM pregnancies with respect to the presence of maternal cervical HPV infection at the time of admission.

Characteristic	with cervical HPV infection (n = 24)	without cervical HPV infection (n = 76)	*p-*value
Respiratory distress syndrome	3 (13%)	11 (15%)	1.00
Transient tachypnea of the newborn	2 (8%)	3 (4%)	0.59
Need for intubation	1 (4%)	2 (3%)	0.57
Intraventricular hemorrhage grade 1–2	3 (13%)	5 (7%)	0.39
Retinopathy of prematurity	1 (4%)	2 (3%)	0.57
Early onset sepsis	0 (0%)	4 (5%)	0.57
Late onset sepsis	0 (0%)	3 (4%)	1.00
Bronchopulmonary dysplasia	2 (8%)	6 (8%)	1.00
Compound neonatal morbidity	8 (33%)	19 (25%)	0.44

Abbreviations:

HPV: human papilloma virus

PPROM: preterm prelabor rupture of membranes

Categorical variables were compared using the Fisher’s exact test and presented as number (%).

Compound neonatal morbidity was defined as a need for intubation, respiratory distress syndrome, transient tachypnea of the newborn, pneumonia, bronchopulmonary dysplasia, retinopathy of prematurity, intraventricular hemorrhage, necrotizing enterocolitis, early onset sepsis, late onset sepsis, and/or neonatal death before hospital discharge.

Necrotizing enterocolitis (n = 1), neonatal death before hospital discharge (n = 1), intraventricular hemorrhage grade 3–4 (n = 0), and pneumonia (n = 0) were not considered in the analysis because of their low occurrence in the cohort.

## Discussion

HPV is a small, double-stranded DNA virus with a circular genome. About 90% of all HPV cervical infections are cleared within 2 years [38, 39]. HPV cervical infection represents the most common cervical viral infection in pregnant women; however, it is likely that HPV is even more common in pregnancies complicated by PPROM [15–17, 40]. Despite this, the role of HPV cervical infection in the pathophysiology of intra-amniotic infection-related and inflammatory complication in PPROM and subsequent development of short-term neonatal morbidity has not been elucidated to date.

The following principal findings were obtained in this study: i) the rate of cervical HPV infection at the time of admission in women with PPROM between 24+0 and 36+6 weeks was 24%, ii) the presence of cervical HPV infection at the time of admission was not associated with a higher rate of infection-related and inflammatory intra-amniotic complications, and iii) cervical HPV infection at the time of admission in women with PPROM was not related to worse short-term neonatal morbidity.

The overall reported rates of HPV cervical infection in pregnant women has been inconsistent in the literature, mainly due to considerable differences in HPV detection methods and various clinical and demographical factors of the cohorts [16, 17, 41–69]. However, the data available for HPV cervical infection in PPROM are limited. In this study, with evaluation for 21 HPV types, the presence of HPV cervical infection at the time of admission was identified in 24% of women with PPROM, and three-fourths of HPV cervical infections were caused by just by 1 HPV type. All women with HPV cervical infection had no clinical symptoms of HPV infection. In spite of the fact that the presence of a high-risk HPV cervical infection has been shown to be related to PPROM [40], the prevalence of cervical HPV infection found in this study was almost the same as (24.6%) in a study from Austria performed in women with gestational ages between 10 and 31 weeks [40]. In addition, the prevalence of HPV cervical infection in our study was similar to the prevalence (28%) revealed in a USA study on healthy women in the third trimester [60].

Recently, cervical infection in pregnant mice caused by murine herpesvirus 68 has been shown to enhance ascent of bacteria from the vagina through the cervix in the uterine cavity, decidua, and amnion [12]. In addition, infection of the placenta caused by the same virus has been related to hyperresponsiveness to low concentrations of bacterial endotoxins, leading to a stronger inflammatory response and preterm birth [13]. Given that HPV is the most common cause of viral cervical infection in pregnancy [16, 17, 41–73], we hypothesized that cervical HPV infection, might be associated with a higher risk of MIAC and subsequent development of IAI. However, in this study, we did not identify any differences in the rate of MIAC and IAI between women with and without HPV cervical infection. We can only speculate whether this lack of the association is driven by the fact that HPV belongs to a different family of viruses (Papillomaviridae) than murine herpesvirus 68. This virus belongs to the Herpesviridae family, which includes other viruses, such as human herpesvirus 6 and 7, herpes simplex virus, cytomegalovirus, and Epstein-Barr virus, which can cause cervical infection in pregnant women [74]. In addition, we did not find any associations between a specific type of HPV and the presence of intra-amniotic infection-related and inflammatory complications. On the other hand, cervical HPV infection caused by more than 1 HPV type tended to show a higher rate of MIAC and IAI, as well as higher amniotic fluid IL-6 levels than HPV cervical infection caused by only 1 HPV type. Given the small sample size (n = 6) of the subgroups of women with 2 or more HPV types, these findings should be interpreted with caution.

A solid body of evidence suggests that viral infection in pregnancy can affect the fetus in various ways: i) by transplacental passage, which may have detrimental and destructive effects on fetal development [74–76], and ii) by extensive inflammatory response in the infected placenta, even when virus does not cross the placental barrier [13]. HPV has been shown to be able to cross the placental barrier and reach the fetus and cord blood [77] and to induce placental infection [46, 52, 61, 65, 68, 77–79]. This study did not find HPV placental infection in the subgroup of women with HPV cervical infection at the time of admission. Therefore, as expected, no differences in the selected aspects of short-term neonatal morbidity between newborns from PPROM pregnancies with and without HPV cervical infection were identified. Given the relatively high rate of HPV cervical infection at the time of admission in pregnancies complicated by in PPROM (every fourth woman), the fact that HPV cervical infection was not related to a higher rate of intra-amniotic complications and worse perinatal and neonatal outcomes can be clinically relevant.

The main strength of this study is the use of a very homogeneous cohort of Caucasian women with a thoroughly defined phenotype of spontaneous preterm delivery in a single tertiary referral center. Second, this study contains data regarding cervical and placental HPV infection, intra-amniotic conditions, and short-term neonatal outcomes. Third, MIAC was defined based on the combination of non-cultivation (nonspecific PCR for 16S rRNA and specific PCR for *Ureaplasma* species, *M*. *hominis*, and *C*. *trachomatis*) and cultivation approaches.

There are some limitations in this study. First, transmission of HPV in amniotic fluid was not assessed in the study. However, no amniotic fluid HPV DNA has been found in studies that assessed amniotic fluid samples from the second trimester obtained during invasive diagnostic procedures, or from the end of the third trimester taken during cesarean sections [68, 80, 81]. Second, the presence of HPV DNA was not evaluated in the entire placenta but in only 4 blocks from the placenta. Given a large villous surface, the heterogeneous distribution of HPV DNA throughout the placenta, and the fact that only fractions of the placenta were subjected to HPV analysis, it is possible that our results regarding HPV placental infection are underestimated. In addition, the HPV DNA were evaluated neither in the fresh nor in the frozen placental samples but in the formalin-fixed paraffin-embedded placental samples in this study There is an evidence that formalin fixation may cause extensive DNA damage including cross-linking and fragmentation [82, 83]. Therefore, the kind of placental samples (formalin-fixed paraffin-embedded) used for the identification of HVP DNA in the placental might have affected the rate of placental HPV infection found in this study. Third, the presence of HPV viruses in newborns from PPROM pregnancies with HPV cervical infection, or those with juvenile onset recurrent laryngeal papillomatosis, was not evaluated in this study. Fourth, the results regarding selected aspects of short-term neonatal morbidity between women with and without cervical HPV infection should be taken with caution due to their low incidences in the cohort.

In summary, HPV cervical infection complicates about one-fourth of PPROM pregnancies between gestational ages 24+0 and 36+6 weeks; however, the presence of HPV cervical infection at the time of admission is not related to a higher risk of intra-amniotic infection-related and inflammatory complications or worse short-term neonatal outcomes.

## Supporting information

S1 dataset (XLS)
